# Circadian Rhythmicity and Light Sensitivity of the Zebrafish Brain

**DOI:** 10.1371/journal.pone.0086176

**Published:** 2014-01-22

**Authors:** Helen A. Moore, David Whitmore

**Affiliations:** Centre for Cell and Molecular Dynamics, Department of Cell and Developmental Biology, University College London, London, United Kingdom; Nagoya University, Japan

## Abstract

Traditionally, circadian clocks have been thought of as a neurobiological phenomenon. This view changed somewhat over recent years with the discovery of peripheral tissue circadian oscillators. In mammals, however, the suprachiasmatic nucleus (SCN) in the hypothalamus still retains the critical role of a central synchronizer of biological timing. Zebrafish, in contrast, have always reflected a more highly decentralized level of clock organization, as individual cells and tissues contain directly light responsive circadian pacemakers. As a consequence, clock function in the zebrafish brain has remained largely unexplored, and the precise organization of rhythmic and light-sensitive neurons within the brain is unknown. To address this issue, we used the *period3 (per3)-luciferase* transgenic zebrafish to confirm that multiple brain regions contain endogenous circadian oscillators that are directly light responsive. In addition, *in situ* hybridization revealed localised neural expression of several rhythmic and light responsive clock genes, including *per3*, *cryptochrome1a* (*cry1a*) and *per2*. Adult brain nuclei showing significant clock gene expression include the teleost equivalent of the SCN, as well as numerous hypothalamic nuclei, the periventricular grey zone (PGZ) of the optic tectum, and granular cells of the rhombencephalon. To further investigate the light sensitive properties of neurons, expression of *c-fos,* a marker for neuronal activity, was examined. *c-fos* mRNA was upregulated in response to changing light conditions in different nuclei within the zebrafish brain. Furthermore, under constant dark (DD) conditions, *c-fos* shows a significant circadian oscillation. Taken together, these results show that there are numerous areas of the zebrafish central nervous system, which contain deep brain photoreceptors and directly light-entrainable circadian pacemakers. However, there are also multiple brain nuclei, which possess neither, demonstrating a degree of pacemaker complexity that was not previously appreciated.

## Introduction

Circadian clocks control a vast number of rhythmic biochemical, physiological and behavioural activities. Understanding the influence and regulation of these rhythms by the brain remains a topic of great importance. To date, zebrafish have contributed relatively little to our understanding of the neural basis of circadian biology. The focus of most clock studies in zebrafish has been directed at the examination of circadian oscillators in peripheral tissues, cell lines and early embryos, where direct light responsiveness has been a central issue [1]–[5]. The circadian system in zebrafish is seen as highly decentralized, and although the brain was shown to be globally rhythmic and light responsive in early studies [6], [7], no detailed analysis of clock function within neural structures has been performed. The one exception to this, of course, is the zebrafish pineal gland, where early studies demonstrated its rhythmicity and direct light sensitivity [8], and more recent work has revealed a potentially critical role in regulating locomotor activity [9]. However, the question still remains of how the circadian system is organized within the zebrafish brain. Is it uniformly rhythmic and light sensitive, or are there discreet, localized circadian and light responsive structures?

The central neuronal pacemaker in the mammalian circadian system, the SCN, is anatomically defined in the zebrafish brain. However, its potential function in the zebrafish circadian system is unknown, although there is some evidence that it is not required for the development of circadian rhythms [10]. Robust rhythmicity is a characteristic of all neuronal pacemakers and can be monitored by measuring, either rhythms in electrical activity or daily oscillations in the expression of core clock genes. For example, robust rhythms of *per1* expression have been described not only in cultured mammalian SCN, but also in extra-hypothalamic structures, such as the olfactory bulb and several other brain nuclei [11]. Expression of *per3* is robustly rhythmic in zebrafish under both entrained, LD and free-running, DD conditions [12]; therefore, the availability of a *per3-luciferase* transgenic zebrafish [13], [14] provides an excellent tool with which to explore the complexity of clock organization within the zebrafish brain and determine the extent of both direct light sensitivity and diversity of neuronal pacemaker structures.

Zebrafish tissues, cell lines and embryos contain directly light entrainable circadian pacemakers, and two acutely light responsive genes, *cry1a* and *per2*, have been shown to be critical for this process [3], [4]. However, there has not yet been a detailed analysis of *cry1a* and *per2* expression in the zebrafish brain in response to light stimulation. Consequently, it is not known whether all regions of the brain are directly light responsive. This issue will be explicitly addressed in this study.

*c-fos* expression is frequently used as a marker for neuronal activity, particularly in the mammalian SCN [15]–[20], which can be subdivided into two regions: the retinofugal ventrolateral SCN region that expresses *c-fos* in response to light, and the dorsomedial SCN region, which does not, but shows an endogenous circadian rhythm in *c-fos* levels. A detailed examination of *c-fos* expression throughout the zebrafish brain, both across the circadian cycle and in response to acute light stimulation, has never been performed. Yet such an analysis could provide clues to identifying “SCN-like” neuronal pacemakers within the zebrafish central nervous system. Furthermore, changes in the expression of *c-fos* in response to light may provide further understanding into how light entrains circadian rhythms in the zebrafish brain. This study will therefore analyse brain nuclei throughout the zebrafish Central Nervous System (CNS) to determine whether there are any regions that display robust rhythmicity of the clock gene, *per3*. Furthermore, the light sensitivity of the brain will be examined by exploring the temporal and spatial expression of *cry1a*, *per2* and *c-fos* in response to light stimulation.

## Results

### *Per3* expression in the zebrafish brain

**Figure 1:**
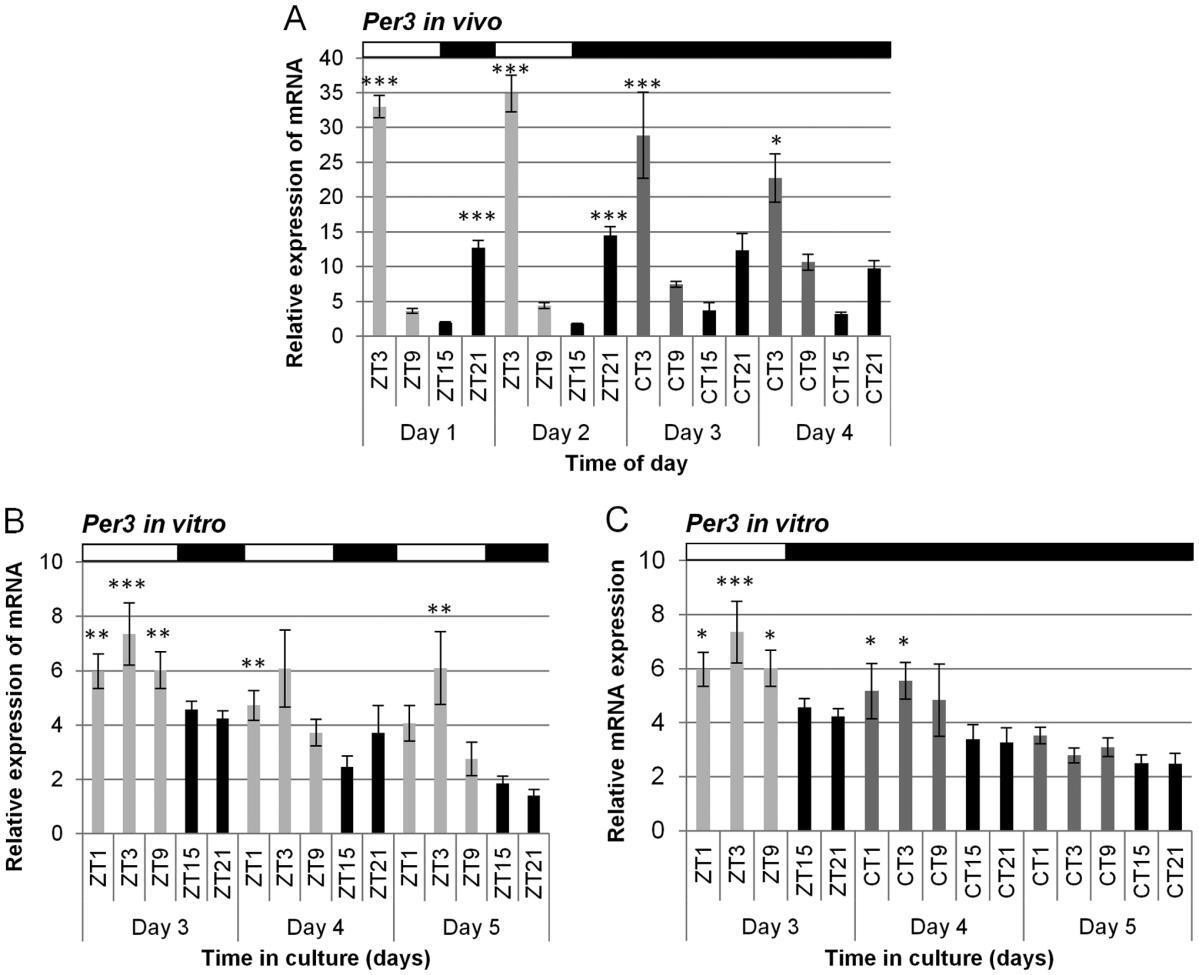
*Per3* is a highly rhythmic circadian clock gene in whole brains *in vivo* and *in vitro.* A) Zebrafish were kept on 2 days of 14:10 LD followed by 2 days of DD, and brains dissected at the times indicated. RNA was extracted and qPCR was performed to evaluate the relative expression of *per3* mRNA. In LD there was a peak at ZT3 and trough at ZT15 (p<0.0001, One way ANOVA, n = 7–10). In DD there was also a peak at CT3 and trough at CT15 (p<0.0001, One way ANOVA, n = 3–5). The statistical significance is shown from the post-hoc Dunnett's multiple comparison test, which used the calibrator day 1 ZT15 for LD conditions, and day 4 CT15 for DD conditions. B) Whole brains were dissected, cultured and kept on a 14:10 LD cycle for 3 days. Samples were collected at the times indicated, RNA extracted and qPCR performed to determine the expression of *per3* mRNA. There was a peak at ZT3, and a trough between ZT15 and ZT 21 (p<0.001, One-way ANOVA, n = 3–4). The statistical significance is shown from the Dunnett's multiple comparison post-hoc test are, using the trough, ZT21, on day 5 as the calibrator. C) Whole brains were dissected as above, cultured, but this time maintained on a 14:10 LD cycle for one day before being placed into constant darkness for two additional days. Samples were collected at the times indicated, mRNA extracted, and qPCR performed to measure the levels of *per3*. The rhythm persisted in *per3* for one cycle under free-running conditions *in vitro* before damping on the second cycle in the dark. The above white black bars represent the lighting conditions, with the different plotted histogram shades representing light, dark or subjective dark phases.

Endogenous expression of *per3* reveals it to be highly rhythmic in the zebrafish brain both *in vivo* and *in vitro* (Figure 1A). The peak of *per3* expression *in vivo*, under a LD dark cycle (14L:10D) is at ZT3, with a trough at ZT15, an approximate 15-fold difference in expression levels (p<0.0001). During two days in constant darkness (DD), the phase remains the same (p<0.0001), although the amplitude falls to approximately 7-fold. When whole adult brain is placed into culture for 5 days on a LD cycle, rhythmic expression of *per3* persists (p<0.001), though not surprisingly, the amplitude of the rhythm is reduced (Figure 1B). The data shown is for the last three days in culture. Interestingly, not only is there no damping of the circadian oscillation, but the phase of the rhythm is retained when under LD conditions, even in the absence of the classical light responsive structures, the eyes and pineal gland. When the whole brain culture is placed into constant darkness (Figure 1C), the rhythm in *per3* can be seen to rapidly damp and is no longer apparent on the fifth day in culture.

**Figure 2:**
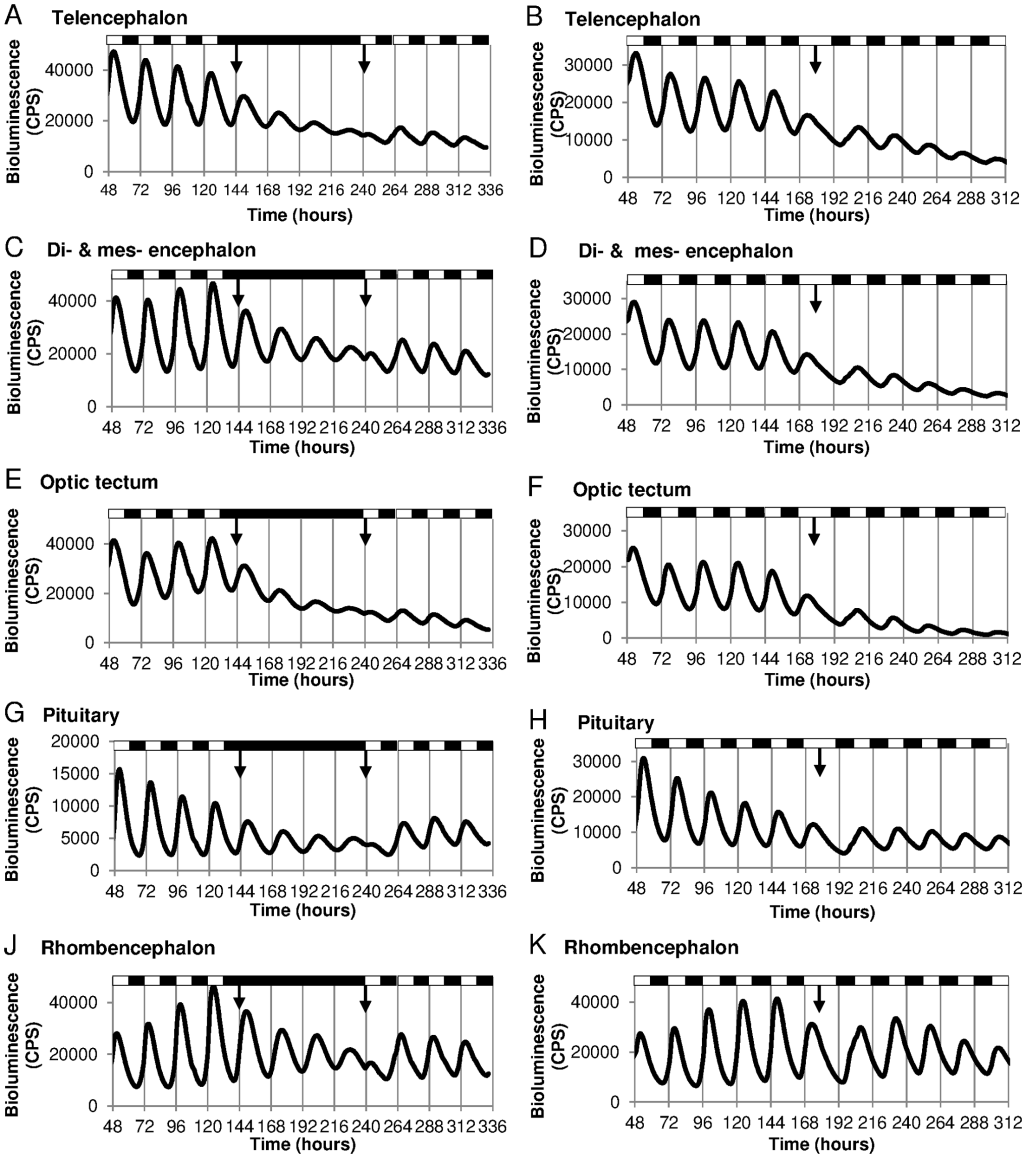
*Per3* rhythms in all isolated regional brain cultures from *per3-luc* zebrafish show entrainment to LD cycles and free-running in DD. Brains were dissected from adult *per3-luc* zebrafish and monitored for bioluminescence in either (A, C, E, G, J) 6 days of 12:12LD, 4 days of DD and 4 days back into LD, or (B, D, F, H, K) 7 days of 12:12LD followed by five cycles of 12:12DL. The mean bioluminescence in counts per second (CPS) is plotted (n = 3–4). All brain regions entrain to a 24-hour period in the LD cycle with a peak around ZT4–6 and free-run with a longer period in DD (p<0.0001, two-tailed paired t-test, n = 3–4 per tissue). All regions rapidly entrain to a new LD cycle or DL cycle with a peak at ZT3-5. Black and white boxes indicate the lighting regime and arrows indicate when this changes.

High temporal resolution recordings of bioluminescent *per3-luciferase* brain cultures reveal that all of the brain regions tested entrain to the LD cycle with a peak between ZT3–5, free run in DD and can re-entrain to a new LD cycle (Figure 2). Five major brain areas were dissected and cultured for 12 days in a 96-well plate while luminescent data was collected. In the left hand traces, these brain regions were exposed to four days of constant darkness in order to examine the free-running characteristics of the *per3* oscillation in these brain regions. For the right hand traces, the LD cycle was reversed on the sixth day in culture, and the re-entrainment of the endogenous clock in these dissected tissues was monitored.

**Figure 3:**
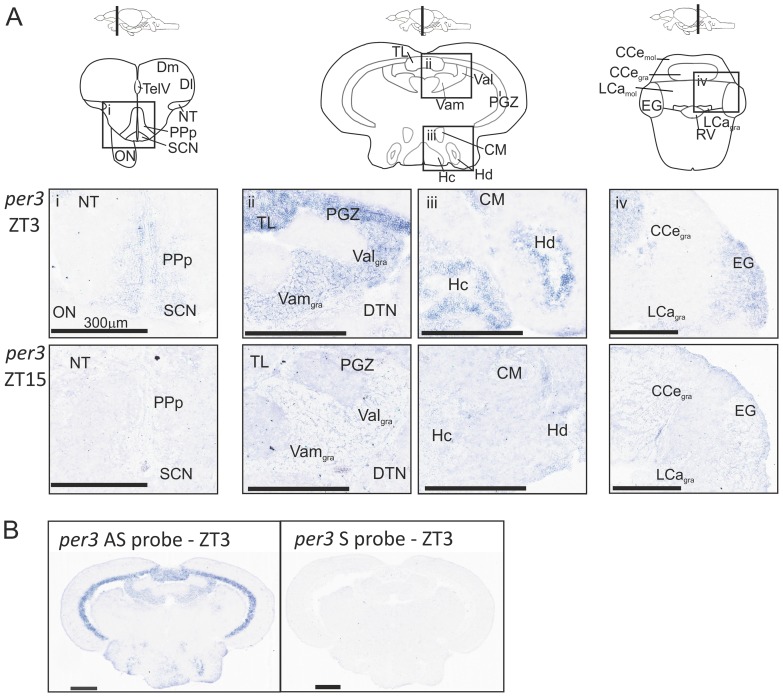
Regional *per3* expression in the adult zebrafish brain. Adult zebrafish were kept on a 14:10LD cycle and brains were collected at ZT3 and ZT15. *In situ* hybridization was performed to show expression of *per3* mRNA. A) Schematics of the brain containing the diencephalon, mesencephalon and rhombencephalon are shown. At ZT3 there is expression of *per3* in the i) PPp and SCN, ii) PGZ, TL, Val_gra_ and Vam_gra_, iii) CM, Hc and Hd, iv) EG, CCe_gra_, and LCa_gra_. At ZT15 there is either low or undetectable levels of *per3* in these same regions. B) The antisense (AS) probe shows the *per3* expression and the sense (S) control shows the background signal.

A careful look at the spatial expression of *per3* by *in situ* hybridisation (ISH) reveals distinct differences between brain nuclei (Figure 3, Table S1). There are many regions where *per3* is highly expressed at ZT3, including the SCN, PPp, PGZ, TL, Val_gra_, Vam_gra_, CM, hypothalamic nuclei (Hc, Hd), EG, CCe_gra_, and LCa_gra_. However, there are many other nuclei that do not express *per3* at a detectable level, including cells in other layers of the optic tectum, LLF, numerous tegmental nuclei (DTN, EW, NI, SGN) and the pretectum. The high levels of *per3* expression in the PGZ and Val can be seen in Figure 3B at ZT3 at the macroscopic level. The use of a sense RNA probe confirms that none of this apparent signal is due to non-specific binding of the *per3* probe.

### Expression of light responsive genes, *cry1a* and *per2*, in the zebrafish brain

**Figure 4:**
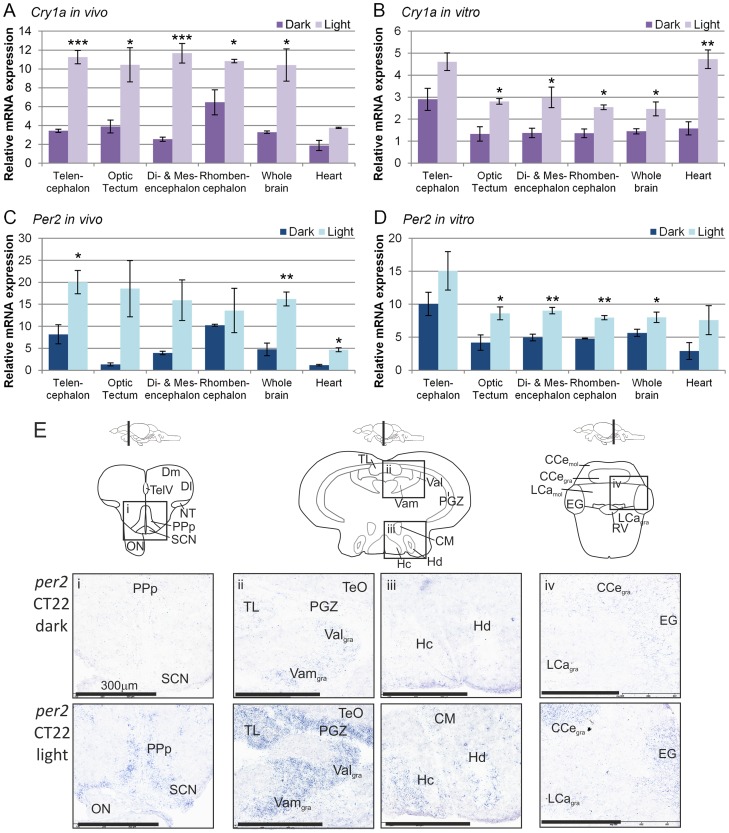
Expression of light sensitive genes, *cry1a* and *per2*, in the adult zebrafish brain. A & C) Wild type adult zebrafish were kept in the dark for 3 days and then either exposed to light for 3 hours or remained in the dark. The zebrafish were killed at CT22 and their brain and hearts dissected, RNA extracted and qPCR performed. The majority of samples collected from different brain regions showed the light responsive genes, *cry1a* and *per2*, were increased in light pulsed tissues compared to dark control. Numerous brain parts showed an increase in *cry1a* and *per2* in the light pulsed samples (p<0.0001 and p = 0.0015 respectively, Two way ANOVA, n = 3). The light pulsed zebrafish had significantly higher c*ry1a* and *per2* levels in both the heart and brain (p<0.002, Two way ANOVA, n = 3). B & D) Wild type adult zebrafish brain parts, whole brains and hearts were cultured in L15-media for four days. Samples were exposed to a 3 hour light pulse or kept in the dark and collected at CT22, RNA extracted, and qPCR performed. *Cry1a* and *per2* is induced by light in both the brain and heart (p<0.0001, Two way ANOVA, n = 3–5). The light pulsed brain part cultures had significantly higher *cry1a* and *per2* levels than the dark controls (p<0.0001 and p = 0.0013 respectively, Two way ANOVA, n = 3). E) Wild type adult zebrafish were kept in the dark for 3 days and then either exposed to light for 3 hours or remained in the dark. The zebrafish were killed at CT22 and their brain dissected, fixed, frozen and sectioned. Chromogenic *in situ* hybridisation was performed to determine the location of *per2* mRNA expression. There was minimal or undetectable expression in the dark samples. In the light pulsed samples expression of *per2* was increased in the i) PPp and SCN, ii) PGZ, TL, Val_gra_ and Vam_gra_, iii) Hc and Hd, iv) EG, CCe_gra_, and LCa_gra_.

Two genes, *cry1a* and *per2,* are believed to be critical for the light entrainment of the zebrafish circadian pacemaker. Expression of both of these genes is increased by an acute three-hour light pulse given to either whole zebrafish or to cultured brains, or dissected areas of the brain in the night (Figure 4A–4D). For comparison, the light induced expression of both genes was also measured in the zebrafish heart. Examination of the spatial expression of *cry1a* and *per2* by *in situ* hybridization reveals a localised increase in expression (Figure 4E, Table S1). There is a considerable overlap between regions that expressed both genes. As with *per3* expression, *per2* was found in the SCN, PPp, PGZ, TL, Val_gra_, Vam_gra_, hypothalamic nuclei (Hc, Hd), EG, CCe_gra_, and LCa_gra_. However, not all areas are light responsive with, for example, *per2* not induced by light in the CM. The *in situ* hybridization sense probe control data for *per3*, as well as the light induced genes and *c-fos*, can be seen in Figure S1. None of these probes produce significant artifactual background staining.

### *c-fos* expression in the zebrafish brain

**Figure 5:**
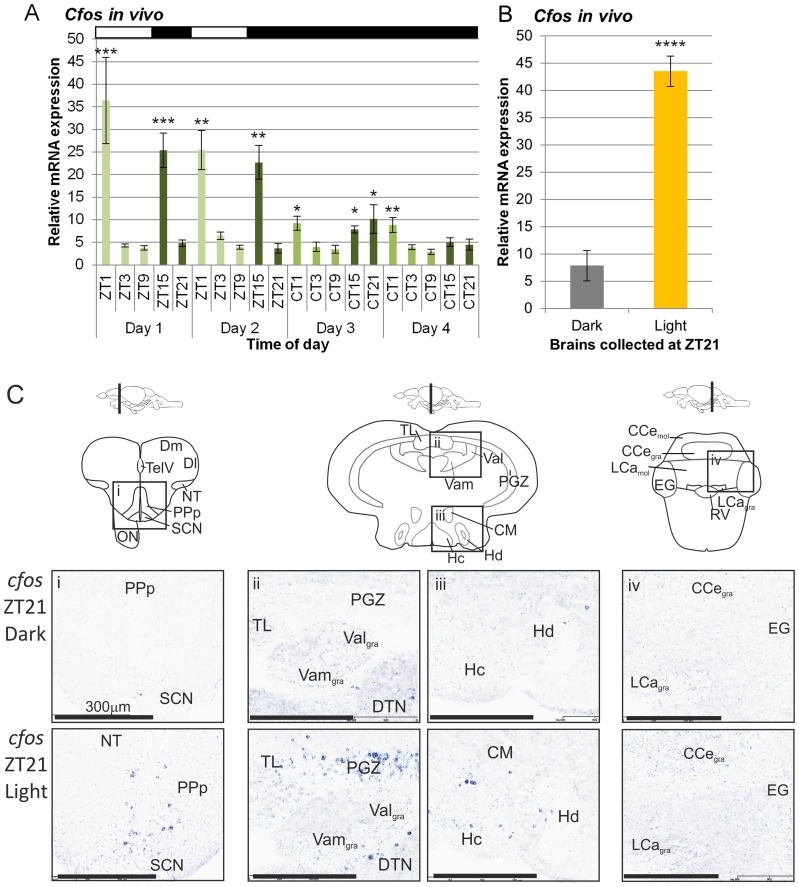
Expression of *c-fos* in the adult zebrafish brain. A) Zebrafish were kept on 2 days of 14:10 LD followed by 2 days of DD, and brains dissected at the times indicated. RNA was extracted and qPCR was performed to evaluate the relative expression of *c-fos* mRNA. In LD there was a peak at ZT1 and ZT15 on day 1 and 2, (p<0.0001, One way ANOVA, n = 4–9). The statistical significance is shown from the post-hoc Dunnett's multiple comparison test, which used the calibrator Day 1 ZT9. In DD there was a peak at CT21 on day 3 and trough at CT9 on day 3 and day 4 (p<0.001, One way ANOVA, n = 4–9). The statistical significance is shown from the post-hoc Dunnett's multiple comparison test, which used the calibrator CT9 on day 4. The above white and black bars indicate the lighting schedule, and the shades of green reflect the light, dark and subjective dark phases. B) Adult zebrafish on a 14:10LD were given a 30 minute light pulse or kept in the dark at ZT21. Brains were dissected, RNA extracted, and qPCR performed to determine levels of *c-fos* mRNA as an indicator of neuronal activity. *C-fos* expression was five-fold higher in the brains of the light pulsed zebrafish (p<0.0001, unpaired two-tailed t-test, n = 7–8). C) *c-fos* is induced in specific brain regions in response to a light pulse in the night. Adult zebrafish maintained on a 14L:10D LD cycle were exposed to a 30-min light pulse at ZT21 or kept in the dark. *In situ* hybridisation was performed on brain sections to determine the levels of *c-fos* mRNA. Regions that show increased *c-fos* expression in response to light include i) PPp and SCN, ii) TeO, TL, Val_gra_ and Vam_gra_ and iii) Hc and Hd. iv) There is no change in expression in the rhombencephalon. Abbreviations: PPp (dorsal pretectum), SCN (suprachiasmatic nuclei), TeO (optic tectum), TL (torus longitudinalis), Val_gra_ (lateral valvula cerebelli), Vam_gra_ (medial valvula cerebelli), Hc (caudal hypothalamus) and Hd (dorsal hypothalamus).

*c-fos* expression *in vivo* in whole adult brain for two days on a light-dark cycle shows a very robust change at both lights-on and lights-off (Figure 5A). Samples collected one hour after dawn show a strong increase in the light (p<0.0001, n = 4–9). There is another strong induction of *c-fos* an hour after lights-off (p<0.0001, n = 4–9). When the animals are allowed to free-run into constant darkness for two additional days, this *c-fos* light response is lost, but a clear circadian rhythm in expression now becomes apparent (p<0.0001, n = 4–9). A 30-minute light pulse during the late night will induce *c-fos* expression strongly compared to dark controls (p<0.0001, n = 7–8), a result similar to that seen in the mammalian SCN in response to phase shifting light pulses. This spatial expression of *c-fos* was further explored for this acute light induction in the night and revealed that *c-fos* was light-induced in specific brain nuclei, including the SCN, PPp, PGZ, TL, Val_gra_, Vam_gra_, and hypothalamic nuclei (Hc, Hd). However, no expression was found in the rhombencephalic nuclei.

## Discussion

In this study, we have performed the first detailed examination of localised rhythmic and light sensitive clock gene expression in the adult zebrafish brain. These areas may represent important regulatory regions of the zebrafish circadian timing system and are potential neuronal pacemakers.

The clock gene *per3* shows robust oscillations in the whole zebrafish brain when dissected at time points under both entrained, LD and free-running, DD conditions. The phase of the rhythm on a LD cycle closely matches that previously described for zebrafish peripheral tissues dissected from the same transgenic fish line [13], [14]. When the whole adult brain is placed into cell culture on a LD cycle for a total of five days, a continuation of this *per3* rhythm is observed. The amplitude of the oscillation *in vitro* is, not surprisingly, significantly reduced compared to the *in vivo* situation. However, this amplitude does not decline across the three LD cycles examined in culture, nor is there any alteration in the phase of entrainment. In constant darkness, rhythmic expression of *per3* persists *in vitro*, but with significant damping, particularly by the second day of DD. Despite these differences in amplitude, these results confirm that the whole brain not only contains an endogenous circadian oscillator, but also is directly light-responsive, as has previously been described for zebrafish peripheral tissues [1], [14].

Are circadian pacemakers found throughout the zebrafish brain, and can the clocks within different regions be directly entrained by a light-dark cycle in culture? Following on from the initial description of light-responsive, peripheral circadian clocks in zebrafish, it has been generally assumed by some groups that the fish brain might function as a unified, rhythmic and light sensitive structure. However, this issue has never been examined in detail. Therefore, we took advantage of the *per3-luciferase* transgenic zebrafish, generated by Cahill and colleagues, and previously employed to explore clock oscillations in peripheral tissues [13], [14]. Various brain regions from this animal were dissected and placed into culture. Bioluminescent rhythms in gene expression were then measured from these tissues for 11 days in culture. All five areas of the brain examined in this way, ranging from the telencephalon, optic tectum to the pituitary gland, showed robust *per3* oscillations that not only persisted for a period of 4 days in DD, but also were able to re-entrain directly to a reversed LD cycle in the middle of the experimental procedure. Each of these brain regions, therefore, is directly light responsive and contains an endogenous clock that is functionally re-entrainable without any light input from the eyes or pineal gland.

The brain areas analysed above still represent quite large neural regions, and so to explore in more detail whether discreet areas of the brain might contain circadian pacemakers, we examined *per3* expression in the adult zebrafish brain using *in situ* hybridization. Numerous nuclei within the brain showed robust rhythmicity in *per3* expression, including the teleost equivalent of the SCN, the paraventricular organ (PVO) (PVN in mammals), the PGZ of the optic tectum, and multiple hypothalamic nuclei. A summary of these results can be found in Table S1. The strong neuronal cell body staining within these brain nuclei can be clearly seen in Figure 3. It is hard to make meaningful comparisons, based on anatomy, between clock function in zebrafish and the well-characterized mammalian brain. However, several of these structures identified above in zebrafish, such as the SCN and PVN, are central to mammalian clock function, as well as the processing of visual or light information. Clearly, considerable future work is required to determine the functional roles of these brain nuclei in zebrafish, and especially the influence that possessing an endogenous circadian pacemaker may have on neural processing in general. Though there is widespread *per3* expression within the zebrafish brain, of considerable interest is the fact that there are brain nuclei lacking apparent expression at either ZT3 or ZT15. This is especially true of the pretectum, including the superficial, central, accessory and posterior pretectal nuclei (PS, CPN, APN and PO). This is somewhat surprising, as this region in mammals is one of the areas to which the recently discovered intrinsically-photosensitive retinal ganglion cells (ipRGCs) are known to project. However, this innervation may be critical for the light-activated pupillary response, a response that is missing in teleosts. In addition, numerous areas of the tegmentum appear to lack *per3* expression, as well as some regions of the cerebellum and the inferior and superior colliculus. It is clear that the distribution of potential circadian oscillators in the zebrafish brain is not as uniform as initially predicted, as shown here by *per3* expression. This result raises the distinct possibility that there are non-rhythmic and even non-clock-containing regions of the adult zebrafish brain, a conclusion that goes somewhat against the simplistic view of zebrafish as being “globally rhythmic”. It must be noted that the regions that did not appear to express *per3* may be due to the cells expressing the transcript at a low level that could not be detected using the ISH method. A more sensitive method, such as qPCR, may detect mRNA, if these regions could be accurately dissected. Alternatively, brain slice luminescent imaging of the *per3-luciferase* transgenic could prove to be a valuable approach. Previous ISH studies in adult zebrafish had noted that rhythmic expression of the clock genes, *clock* and *bmal*, was, in fact, restricted to certain brain areas [6], [7], but this analysis was quite limited and not extended to the entire brain. Upon re-examination and re-evaluation of this previous data, it is encouraging that there are a number of brain regions, including the PGZ, Val (cerebellum) and hypothalamic nuclei, that express both *clock* and *bmal*, as well as *per3* shown in this current study. Additionally, a recent study examined the expression of several clock genes in adult zebrafish brain and found similar results [21].

Light entrainment of the zebrafish clock within cell lines, embryos and tissues appears to require the acute induction of *cry1a* and *per2* [3], [4]. The expression levels of both genes were therefore examined in the brain in response to a three-hour light pulse given in the late subjective night *in vivo* and *in vitro*. Both genes were strongly induced by light in multiple regions of the zebrafish brain *in vivo*. Perhaps more interestingly, their expression was also strongly induced in these same regions under organ culture conditions, when neither the eye nor pineal was present. This response, of course, is likely to underpin the autonomous re-entrainment of the circadian clock shown in *per3* luminescent brain regions in Figure 2.

A closer examination of light-induced expression of *cry1a* and *per2* was performed by *in situ* hybridization. These results are summarized in Table S1. In many cases, specific regions of the brain that show clear *per3* rhythmicity also demonstrate a distinct, acute induction of *cry1a* and *per2*, suggesting that these regions of the brain contain directly entrainable circadian clocks. Curiously, there are some regions of the brain, which appear to be light responsive, but not rhythmic and vice versa. For example, the light responsive clock genes were expressed in the periventricular nucleus of the posterior tuberculum (Tpp), the dorsal posterior thalamic nucleus (DP) and central posterior thalamic nucleus (CP), but these regions did not express *per3*. This suggests that these areas can detect light, but might not possess a functional circadian clock. Furthermore, the anterior tuberal nucleus (ATN), the dorsal zone of D (Dd), the entopeduncular nucleus (EN) and torus lateralis (TLa) expressed *per3*, but not *cry1a* and *per2*. This raises the rather fascinating question of how these particular areas might entrain to light, and whether this occurs directly or due to innervation from other neural areas or even possibly from the retina or pineal gland. There are several regions of the brain in which neither *cry1a*, *per2* nor *per3* can be detected, including many nuclei in the pretectum, tegmentum and brainstem. This result suggests that these areas are neither clock containing nor directly light sensitive. It should be noted, however, that there are multiple genomic replications of certain clock genes in zebrafish, and so at this time, there is no definitive evidence to say these regions are circadian clock-free. Furthermore, there is the possibility of different phase relationships of gene expression in certain nuclei, which would have been missed with the temporal resolution of these studies. As always, negative data needs to be interpreted with some caution. Nonetheless, the expression of only certain clock genes in some regions hints at the fine-tuning of circadian regulation in the zebrafish brain. These data, so far, suggest that a network of multiple neuronal pacemakers and light responsive regions exist within the adult zebrafish brain. However, the behavioural and neurophysiological meaning of these results is not clear, and will require extensive future examination.

Why do zebrafish express high levels of *per3*, *cry1a*, and *per2* in specific regions, rather than uniformly throughout the brain? One suggestion is that these particular regions are receiving light input from the retina, as with the mammalian SCN. Indeed, the clock genes are expressed in many regions that are reported to receive retinofugal inputs in zebrafish, including the ventral thalamic nuclei, PGZ, and Vd [22]. However, not all regions that receive input from the retina express these clock genes, for example, pretectal regions, such as the central pretectal nuclei (CPN). Furthermore, there are many regions that express clock genes that have not been reported to be receive retinofugal inputs in teleosts, such as the hypothalamus, TL, preglomerular nuclei, and valvula cerebelli [23]. Therefore, it appears that retinofugal inputs are not necessary for clock gene expression, and in reality *in vivo*, there is probably a combination of light input from classical sensory structures (eyes and pineal), as well as direct neuronal light sensitivity.

It has been suggested that the localised expression of clock genes could be due to the presence of particular neurotransmitters. Dopamine, and numerous other neurotransmitters, plays a major role in the mammalian SCN, and it is plausible that the regions expressing clock genes could share the same neurotransmitter cell types. [24]. Tyrosine hydroxylase, a key enzyme in the synthesis of dopamine, has been used to label dopamine cells throughout the zebrafish [25]. Certainly, dopamine cells can be found in regions that express clock genes, including the SCN, Hc, Hv, PPa and Vd. However, dopamine cells are not located in many other regions expressing clock genes, including the PG, Hd and PGZ. These regions instead express many other neurotransmitter cell types, for example the PGZ consists of serotonergic [26], GABAergic [27] and adrenergic cells [28]. Therefore, the regional expression of clock genes in zebrafish is not due to the presence a particular neurotransmitter cell type.

One potentially interesting correlation regarding the localisation of clock gene expression is with the hypocretin/orexin system. Zebrafish express one hypocretin receptor (hcrtr), which responds to the wake promoting neuropeptide hypocretin (hcrt) [27]. The hcrt/orexin system is, of course, involved in sleep regulation and arousal in numerous species. In zebrafish, there appears to be some overlap with regions that express *hcrtr* and *per3*, including the ventral thalamic nuclei, PGZ, hypothalamus, and the cerebellum. However, there are also a few notable differences, including the expression of *hcrtr* in the griseum centrale, which does not express *per3*. However, the relationship between the expression of clock genes and the sleep-regulatory regions of the zebrafish brain is certainly worthy of further examination, as it is feasible that the robust expression of clock genes, as well as direct light sensitivity, in regions regulating sleep could be key to the regulation of this process.

The rapid induction of *c-fos* expression is often used as a marker of neuronal activity and has been characterised in various mammalian neural [15]–[20,] and retinal tissues [20], [29] in response to circadian light input and rhythmicity within the SCN. An examination of *c-fos* expression in the whole adult brain on both a LD cycle and in DD revealed quite a complex, but precise level of regulation. When animals are exposed to a LD cycle, *c-fos* expression changes rapidly following both the lights-on and lights-off signal. One hour after both dawn and dusk, there is a strong peak in *c-fos* induction, marking the transition in lighting conditions, a response that is no longer present when animals are placed into DD. Under constant dark conditions, however, an apparent circadian oscillation in *c-fos* expression is revealed, which peaks in the late subjective night. This rhythm appears to damp quite rapidly, showing a significant loss of amplitude on the second day in DD. A 30-minute light pulse given to fish in the late night (ZT21) causes a strong induction in *c-fos* levels in whole brain samples. *In situ* hybridization for *c-fos* reveals that the light sensitive regions are found throughout the zebrafish brain, including many regions of the hypothalamus. This is consistent with the examination of deep brain photoreceptors in other species [30]. It is apparent that there are multiple points of regulation for *c-fos* in the zebrafish brain. Clearly, there is a response to changing light conditions, with a strong response to both lights on and off, which may represent a distinct change in neural activity that occurs to alterations in sensory input at these moments. Secondly, there is a level of endogenous clock regulation for *c-fos*, which may represent daily rhythms in neural activity in certain brain regions. Finally, there is an acute induction of *c-fos* in response to light stimulation in the night, as has previously been described in mammalian clock studies [15], [17]. The biological consequences and role of these changes is far from clear, but is a major topic for future examination.

The results presented in this study, especially those relating to *c-fos* induction and to *cry1a* and *per2* induction by light, raises many questions about the nature of this direct neural light sensitivity. It is possible that the zebrafish brain contains numerous types of photosensitive cells, each of which may contain either the same or, of course, different photopigments. Numerous photopigments have been put forward as candidates for the light responsive component of the zebrafish clock, including opsins, cryptochrome4 and flavin-containing oxidases [31]. However, a detailed expression analysis within the zebrafish brain is critical if the role of single or multiple photopigments in zebrafish clock entrainment is to be determined.

Evidence for direct brain light sensitivity in zebrafish has been growing in recent years, even in aspects of biology not directly related to circadian clock entrainment. Several opsins have already been described, which show expression patterns within specific regions of the brain (as mentioned above), and provide a potential mechanism for the light responses described in this manuscript. These include Vertebrate Ancient-Long (VAL) opsin in the diencephalic ventricle of the central thalamus, as well as specific expression for various melanopsin isoforms within the brain [32]–[34]. Zebrafish behaviour is also altered in response to light stimuli in animals lacking both eyes and the pineal gland. One particular “dark photokinesis” response has been associated with the expression of melanopsin (opn4a) in the preoptic area of the brain [35]. In addition, a fast light-induced photomotor response (PMR) is activated in eyeless and pineal-less zebrafish, and this response has been correlated to light-regulated calcium changes within the hindbrain of these individuals [36]. It is becoming clear that this neural direct light sensitivity may have wide ranging behavioural consequences for these animals, in addition to direct neural circadian clock entrainment.

In conclusion, this study has provided a detailed examination of rhythmic and light sensitive clock gene expression in the zebrafish brain, showing that it has the potential to contain numerous neuronal circadian pacemakers. This study has gone some way towards enhancing our understanding of the regulation of circadian rhythms in the zebrafish brain, has demonstrated that it may not be the uniform circadian structure initially assumed, but that equally it is not “SCN-dominated”, and has opened up new possibilities for further investigation.

## Materials and Methods

### Fish care

Wild type (AB/TL) and *per3-luciferase* transgenic zebrafish (*Danio rerio*) were raised in the University College London Fish Facility according to standard procedures [37]. All animals were maintained in a Home Office approved facility, and handled in accordance with the Animal Welfare Act of 2006. Animals were killed in accordance with Schedule 1 procedures of the Home Office Act. All experiments were performed under animal license number PIL70/20002.

For *in vivo* experiments, adult zebrafish were housed in light-tight cabinets at 28°C and exposed to a LD cycle of 14 hours light, 10 hours dark (14L:10D) and then transferred into DD for the following 2 days. Whole brain was dissected from at least 3 animals per time point and harvested in TRIzol (see below) every 6 hours for up to 4 days (2 days of LD into 2 days of DD). For light pulse experiments, animals were transferred into DD and light pulsed for 30 min or 3 hours. Whole brain or brain parts were dissected from at least 3 animals per condition (light pulsed and dark controls). Tissue samples were harvested in TRIzol (Life Technologies) and processed as described below.

### Brain organ culture

Whole brain or brain parts were dissected from adult zebrafish in sterile PBS containing penicillin/streptomycin (100 U/ml). These tissues were placed into a 35 mm petri dish and cultured in Leibovitz's L-15 media without phenol red (Life Technologies), containing 15% fetal calf serum (Biochrom AG), penicillin/streptomycin (100 U/ml) and gentamicin (50 µg/ml) (Life Technologies). Dishes were sealed with parafilm and maintained at 28°C on a LD cycle (14L:10D) for up to 5 days or transferred into DD after 3 days of LD. Samples were collected at the times indicated in the figure legends from days 3 to 5 of culture. For light pulse experiments, tissues were maintained on a LD for 2 days, transferred into DD and exposed to light for 3 hours at CT19 of the following night. Light-pulsed and dark control samples were harvested at CT22 as above in TRIzol.

#### Light pulsing procedure

For all luminescent experiments, tissues and brain regions were placed into 96 well plates, which are then illuminated laterally by a white LED light source (400–700 nm). Typically samples were maintained on a 12 hour light –12 hour dark schedule, with the intensity of light at approximately 2000 μW/cm^2^. However, the intensity of light reaching the bottom of the wells on the plate would be significantly lower.

Light pulses applied to whole fish and to brain regions in culture were also “white” light signals (400–700 nm) at an intensity of 800 μW/cm^2^, for durations specified with each experiment.

### RNA extraction and quantitative PCR

Total RNA was isolated from dissected, or dissected and cultured, brains using TRIzol Reagent (Life Technologies), following the manufacturer's instructions, and cDNA was synthesized from 0.5 μg to 2 μg of total RNA and SuperScript II Reverse Transcriptase (Invitrogen). The quantitative PCR (qPCR) reaction was carried out in a Mastercycler ep Realplex^2^ (Eppendorf) using SYBR Green Jumpstart Taq Ready Mix (Sigma) and 0.5 μM of gene-specific primers listed below. ΔCt was determined using rpl13a as a reference gene, which was extremely constant in expression levels across all samples tested. Relative expression was calculated using the ΔΔCt method. Results from rhythmic experiments were analysed using a one-way analysis of variance, followed by a Dunnett's multiple comparison post-test. Results from light pulse experiments were analysed using a two-tailed unpaired Student's t-test. P values less than 0.05 were considered significant.

The qPCR primers employed in this study include *rpl13a* (5′-TCTGGAGGACTGTTAGAGGTATGC; 3′-AGACGGACAATCTTGAGAGCAG), *cry1a* (5′-TCCAACCCTAATGGAAGCAC; 3′-ACTCCTCGCTGTGTCGTTTT), *c-fos* (5′-CAGCTCCACCACAGTGAAGA; 3′-GCTCCAGGTCAGTGTTAGCC), *per2* (5′-TGGCTCTGGACAGAAGTGAG; 3′-GGATGTCTCGAGAATGCAAC), and *per3* (5′-CAGCAACGATTCCTCAGACA; 3′-GCTTGATCATGCTCCACAGA).

### Bioluminescent assays

Total bioluminescence of brain tissue dissected from *per3-luciferase* transgenic zebrafish was monitored using a Topcount NXT scintillation counter (Packard) at 28°C. Tissues were dissected and placed into 96-well plates in L15 media described above containing 0.5 mM luciferin. The lighting conditions are indicated on the figures and described in the figure legends. To determine period and phase, these values were standardised with *splinefun* function (http://bitly.com/Sfse6r) from the statistics package for R (http://www.r-project.org/). Measurements were smoothed according to a kernel regression using the *npreg* (http://bitly.com/VlENLI) regression from the *np* package for R, with a bandwidth chosen by expectation maximization. Finally, the first derivative points of the signals gave the time of the peak and troughs throughout the recording, from which the period length and phase shifts were measured.

### *In situ* hybridisation

Adult zebrafish brains were fixed for 2 hours at room temperature (RT) in 4% paraformaldehyde (PFA) in 0.1 M phosphate buffer (PB), cryopreserved overnight using 30% sucrose in 0.1 M PB at 4°C, embedded in Tissue-Tek® optimal cutting temperature (OCT) compound (Sakura), and stored at −80°C. Tissue was sectioned at a thickness of 10 μm and either used immediately at RT, or stored at −80°C for up to six months. A standard in situ hybridisation (ISH) protocol was used. Slides were brought to RT, fixed in 4% PFA in 0.1 M PB, washed with PBS, and treated for 5 min with 1 μg/ml proteinase K in 50 mM Tris-HCl, pH 7.5 and 6 mM EDTA. Samples were then re-fixed in 4% PFA in 0.1 M PB, washed with PBS, and acetylated in triethanolamine, hydrochloric acid, and acetic anhydride solution to reduce non-specific binding. Samples were then washed with PBS, pre-hybridised for 1 hour in hybridisation solution [50% formamide, 5X sodium chloride sodium citrate buffer (SSC), 5X Denhardt's, 250 μg/ml Baker's yeast tRNA, and 500 μg/ml salmon sperm DNA], and incubated with the DIG-labelled RNA probe [either anti-sense (AS) or sense (S) control] in hybridisation solution overnight (ON) at 72°C. The following day, samples were washed once in 5X SSC for 5 min and twice in 0.2X SSC for 40 min at 72°C. Slides were then brought to RT in 0.2X SSC and equilibrated in buffer 1 (0.1 M Tris, pH 7.5, 0.15 M NaCl), before blocking in 10% goat serum (Sigma) in buffer 1 and incubating ON at RT with anti-DIG-alkaline phosphatase (anti-DIG-AP) in 1% goat serum in buffer 1. On the final day, slides were washed in buffer 1 and incubated with the AP substrate, nitro-blue tetrazolium chloride (NBT) and 5-bromo-4-chloro-3-indolyl phosphate (BCIP) in buffer 3 (0.1 M Tris-Cl pH 9.5, 0.1 M NaCl, 0.05 M MgCl_2,_ 0.1% Tween-20). Slides were left to develop in the dark for a few hours at RT, or ON at 4°C. Slides were washed with PBS, incubated for 10 min with DAPI (4′,6-diamidino-2-phenylindole), washed with water and mounted with Glycergel Mounting Medium (Dako). Slides were imaged using a NanoZoomer Slide Scanner (Hamamatsu) or a SCN400 Slide Scanner (Leica). Figures were compiled using NDP.view (Hamamatsu) or SCNviewer (Leica) and CorelDRAW (Corel).

## Supporting Information

Figure S1 **Sense controls for brain** ***in situ*** **hybridization experiments.** The panels in this figure show *in situ* hybridization results for brain sections stained with the sense control probes for *per3*, *per2*, and *c-fos* in fore-, mid- and hindbrain regions of the zebrafish brain. In the case of all three probes used and for all of the brain areas examined, no significant staining was detected. The positive staining reported in the previous figures for these areas, therefore, is unlikely to represent an artefact due to non-specific binding or trapped dye.
(TIF)

Table S1 **A summary of all** ***in situ*** **hybridization results.**This table summarizes all of the *in situ* hybridization data collected in this study for *per3* rhythmicity, *cry1a* and *per2* light induction, and *c-fos* changes in response to light for all of the brain regions examined. Strong positive staining is subjectively indicated with “++”, weaker staining as “+”, with no observable staining indicated by a “−”.
(DOCX)
