# Supplementary figures and images for: Circadian Rhythmicity and Light Sensitivity of the Zebrafish Brain
Source: PLoS One. 2014 Jan 22;9(1):e86176. doi: 10.1371/journal.pone.0086176 (PMC3899219; doi:10.1371/journal.pone.0086176)

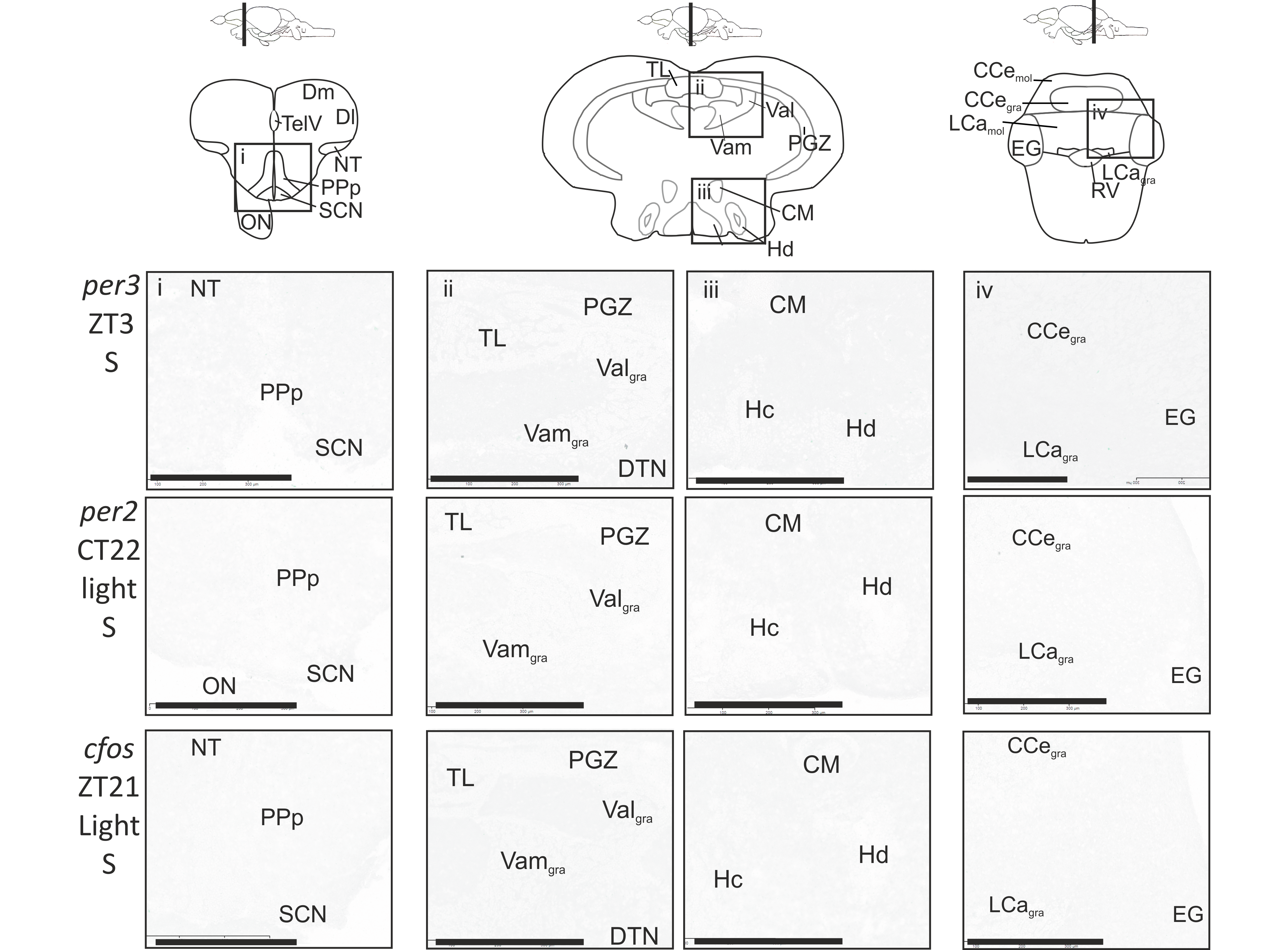

Supplement: Figure S1 — Sense controls for brain in situ hybridization experiments. The panels in this figure show in situ hybridization results for brain sections stained with the sense control probes for per3, per2, and c-fos in fore-, mid- and hindbrain regions of the zebrafish brain. In the case of all three probes used and for all of the brain areas examined, no significant staining was detected. The positive staining reported in the previous figures for these areas, therefore, is unlikely to represent an artefact due to non-specific binding or trapped dye. (TIF) [file pone.0086176.s001.tif]
