# Supplementary material for: Circadian Rhythmicity and Light Sensitivity of the Zebrafish Brain
Source: PLoS One. 2014 Jan 22;9(1):e86176. doi: 10.1371/journal.pone.0086176 (PMC3899219; doi:10.1371/journal.pone.0086176)
Supplement: Table S1 — A summary of all in situ hybridization results.This table summarizes all of the in situ hybridization data collected in this study for per3 rhythmicity, cry1a and per2 light induction, and c-fos changes in response to light for all of the brain regions examined. Strong positive staining is subjectively indicated with “++”, weaker staining as “+”, with no observable staining indicated by a “−”. (DOCX) [file pone.0086176.s002.docx]

| **Zebrafish brain nuclei** | **Circadian genes** | | | | | | **Neuronal activity** | | | |
| --- | --- | --- | --- | --- | --- | --- | --- | --- | --- | --- |
|  | **Rhythmic** | | **Light Responsive (CT22)** | | | | ***C-fos*** | | | |
|  | ***Per3* (ZT15)** | ***Per3* (ZT3)** | ***Cry1a* (D)** | ***Cry1a* (LP)** | ***Per2* (D)** | ***Per2* (LP)** | **ZT3 (L)** | **ZT15 (D)** | **ZT21 (D)** | **ZT21 (LP)** |
| *Telencephalon* |  |  |  |  |  |  |  | | | |
| *OB, Olfactory Bulbs* |  |  |  |  |  |  |  | | | |
| ICL, internal cellular layer | **-** | **++** | **-** | **-** | **-** | **++** | **-** | **++** | **-** | **-** |
| ECL, external cellular layer | **-** | **++** | **-** | **++** | **+** | **+** | **-** | **-** | **-** | **++** |
| GL, glomerular layer | **-** | **-** | **-** | **-** | **-** | **-** | **-** | **-** | **-** | **-** |
| *D, Dorsal telencephalic area* |  |  |  |  |  |  |  | | | |
| Dc, central zone of D | **-** | **-** | **-** | **-** | **-** | **+** | **-** | **-** | **-** | **+** |
| Dd, dorsal zone of D | **-** | **++** | **-** | **-** | **-** | **-** | **-** | **-** | **-** | **+** |
| Dl, lateral zone of D | **-** | **+** | **+** | **+** | **+** | **++** | **-** | **++** | **-** | **+** |
| Dm, medial zone of D | **-** | **-** | **+** | **+** | **+** | **++** | **-** | **++** | **+** | **+** |
| Dp, posterior zone of D | **-** | **-** | **-** | **++** | **-** | **+** | **-** | **-** | **-** | **-** |
| *V, Ventral telencephalic area* |  |  |  |  |  |  |  | | | |
| Cv, commissural nucleus of V | **-** | **-** | **-** | **-** | **-** | **-** | **-** | **-** | **-** | **-** |
| Vd, dorsal nucleus of V | **-** | **++** | **-** | **++** | **-** | **++** | **+** | **++** | **+** | **++** |
| Vv, ventral nucleus of V | **-** | **++** | **-** | **++** | **-** | **++** | **-** | **++** | **-** | **++** |
| Vp, postcommissural nucleus of V | **-** | **++** | **-** | **++** | **-** | **++** | **+** | **++** | **-** | **++** |
| Vs, supracommissural nucleus of V | **-** | **++** | **-** | **++** | **-** | **++** | **-** | **++** | **-** | **++** |
| Vc, central nuclei of V | **-** | **++** | **-** | **++** | **-** | **++** | **-** | **++** | **-** | **++** |
| Vl, lateral nuclei of V | **-** | **-** | **-** | **-** | **-** | **-** | **-** | **-** | **-** | **-** |
| EN, entopeduncular nucleus | **-** | **++** | **-** | **+** | **-** | **-** | **-** | **++** | **-** | **+** |
| NT, nucleus taeniae | **-** | **++** | **-** | **++** | **-** | **-** | **-** | **++** | **-** | **-** |
| *Diencephalon* |  |  |  |  |  |  |  | | | |
| *Area preoptica* |  |  |  |  |  |  |  | | | |
| CO, optic chiasm | **-** | **-** | **-** | **-** | **-** | **-** | **-** | **-** | **-** | **-** |
| OT, optic tract | **-** | **-** | **-** | **-** | **-** | **-** | **-** | **-** | **-** | **+** |
| PPa, parvocellular preoptic nucleus, anterior part | **-** | **++** | **-** | **++** | **-** | **++** | **-** | **++** | **-** | **+** |
| PPd, dorsal part of the pretectal diencephalic cluster | **-** | **++** | **-** | **++** | **-** | **++** | **-** | **++** | **-** | **-** |
| PPp, parvocellular preoptic nucleus, posterior part | **-** | **++** | **+** | **++** | **-** | **++** | **-** | **++** | **-** | **-** |
| SCN, suprachiasmatic nucleus; | **-** | **++** | **-** | **++** | **-** | **++** | **-** | **++** | **-** | **+** |
| *Epithalamus* |  |  |  |  |  |  |  | | | |
| Ha, ventral and dorsal habenula | **-** | **++** | **-** | **++** | **-** | **++** | **-** | **++** | **-** | **+** |
| Pineal | n.d. | n.d. | n.d. | n.d. | n.d. | n.d. | n.d. | n.d. | n.d. | n.d. |
| *Thalamus* |  |  |  |  |  |  |  | | | |
| VM, ventromedial thalamic nucleus | **-** | **++** | **-** | **++** | **-** | **++** | **+** | **++** | **-** | **++** |
| VL, ventrolateral thalamic nucleus | **-** | **++** | **-** | **++** | **-** | **++** | **-** | **++** | **-** | **++** |
| A, anterior thalamic nucleus | **-** | **++** | **-** | **++** | **-** | **++** | **-** | **++** | **-** | **++** |
| DP, dorsal posterior thalamic nucleus | **-** | **-** | **-** | **++** | **-** | **++** | **-** | **++** | **-** | **++** |
| CP, central posterior thalamic nucleus | **-** | **-** | **-** | **++** | **-** | **++** | **-** | **++** | **-** | **++** |
| ZL, zona limitans | **-** | **++** | **-** | **++** | **-** | **++** | **-** | **++** | **-** | **-** |
| *Pretectum* |  |  |  |  |  |  |  | | | |
| PS, superficial pretectal nuclei | **-** | **-** | **-** | **-** | **-** | **-** | **-** | **-** | **-** | **+** |
| CPN, central pretectal nucleus | **-** | **-** | **-** | **-** | **-** | **-** | **-** | **-** | **-** | **+** |
| APN, accessory pretectal nucleus | **-** | **-** | **-** | **-** | **-** | **-** | **-** | **-** | **-** | **-** |
| PO, posterior pretectal nucleus | **-** | **-** | **-** | **-** | **-** | **-** | **-** | **-** | **-** | **-** |
| DAO/VAO, accessory optic nuclei | **-** | **-** | **-** | **-** | **-** | **-** | **-** | **-** | **-** | **-** |
| PP, periventricular pretectal nucleus | **-** | **-** | **-** | **+** | **-** | **+** | **-** | **-** | **-** | **+** |
| *Posterior tuberculum* |  |  |  |  |  |  |  | | | |
| Tpp, periventricular nucleus of the posterior tuberculum | **-** | **-** | **-** | **++** | **-** | **-** | **-** | **++** |  | **+** |
| TLa, torus lateralis | **-** | **++** | **-** | **-** | **-** | **-** | **-** | **-** | **-** | **-** |
| CM, corpus mammilare | **+** | **++** | **-** | **++** | **-** | **-** | **-** | **++** | **-** | **+** |
| PTN, posterior tuberal nucleus | **-** | **++** | **-** | **++** | **-** | **++** | **-** | **++** | **-** | **++** |
| PVO, paraventricular organ | **-** | **++** | **-** | **++** | **-** | **++** | **-** | **++** | **-** | **-** |
| PGl/a, lateral/ anterior preglomerular nucleus | **-** | **++** | **-** | **++** | **-** | **++** | **-** | **++** | **-** | **-** |
| PGm, medial preglomerular nucleus | **-** | **++** | **-** | **++** | **-** | **++** | **+** | **++** | **-** | **+** |
| *Hypothalamus* |  |  |  |  |  |  |  | | | |
| ATN, anterior tuberal nucleus | **-** | **++** | **-** | **-** | **-** | **-** | **-** | **++** | **-** | **-** |
| CIL, central nucleus of the inferior lobe | **-** | **++** | **-** | **-** | **-** | **++** | **-** | **-** | **-** | **-** |
| DIL, diffuse nucleus of the inferior lobe | **-** | **++** | **-** | **-** | **-** | **++** | **+** | **++** | **+** | **++** |
| Hc, caudal zone of periventricular hypothalamus | **+** | **++** | **-** | **++** | **-** | **++** | **-** | **-** | **-** | **++** |
| Hd, dorsal zone of periventricular hypothalamus | **+** | **++** | **-** | **++** | **+** | **++** | **-** | **+** |  | **+** |
| Hv, ventral zone of periventricular hypothalamus | **-** | **++** | **-** | **++** | **-** | **++** | **+** | **++** | **+** | **++** |
| LH, lateral hypothalamic nucleus | **-** | **++** | **-** | **++** | **-** | **++** | **-** | **-** | **-** | **-** |
| *Superior and inferior colliculi* |  |  |  |  |  |  |  | | | |
| TeO, optic tectum | **-** | **+** | **-** | **-** | **-** | **+** | **-** | **-** | **-** | **+** |
| PGZ, periventricular grey zone | **-** | **++** | **-** | **++** | **-** | **++** | **+** | **++** | **+** | **++** |
| TL, torus longitudinalis | **-** | **++** | **-** | **++** | **-** | **++** | **-** | **++** | **-** | **-** |
| TS, torus semicircularis | **-** | **+** | **-** | **-** | **-** | **-** | **+** | **++** | **-** | **-** |
| LLF, lateral longitudinal fascicle | **-** | **-** | **-** | **-** | **-** | **-** | **-** | **-** | **-** | **-** |
| MNV, mesencephalic nucleus of trigeminal nucleus | **-** | **-** | **-** | **-** | **-** | **-** | **-** | **-** | **-** | **-** |
| Nmlf, nucleus of the medial longitudinal fascicle | **-** | **-** | **-** | **-** | **-** | **-** | **-** | **-** | **-** | **-** |
| PCN, paracommissural nucleus | **-** | **-** | **-** | **-** | **-** | **-** | **-** | **-** | **-** | **-** |
| *Tegmentum* |  |  |  |  |  |  |  | | | |
| DTN, dorsal tegmental nucleus | **-** | **-** | **-** | **-** | **-** | **-** | **-** | **++** |  | **++** |
| EW, Edinger-Westphal nucleus | **-** | **-** | **-** | **-** | **-** | **-** | **-** | **-** | **-** | **-** |
| NLV, nucleus lateralis valvulae | **-** | **++** | **-** | **-** | **-** | **-** | **-** | **+** |  | **+** |
| NIII, Oculomotor nucleus | **-** | **-** | **-** | **-** | **-** | **-** | **-** | **-** | **-** | **-** |
| NLL, nucleus of lateral lemniscus | **-** | **-** | **-** | **-** | **-** | **-** | **-** | **-** | **-** | **-** |
| PL, perilemniscal nucleus | **-** | **++** | **-** | **++** | **-** | **++** | **-** | **++** | **-** | **-** |
| SR, superior raphe nucleus; | **-** | **-** | **-** | **-** | **-** | **-** | **-** | **-** | **-** | **-** |
| SRF, superior reticular formation | **-** | **-** | **-** | **-** | **-** | **-** | **-** | **-** | **-** | **-** |
| NIn, nucleus interpeduncularis | **-** | **-** | **-** | **-** | **-** | **-** | **-** | **-** | **-** | **-** |
| NI, nucleus isthmus | **-** | **-** | **-** | **-** | **-** | **-** | **-** | **+** | **-** | **-** |
| LC, Locus coeruleus | **-** | **-** | **-** | **-** | **-** | **-** | **-** | **-** | **-** | **-** |
| SGN, secondary gustatory nucleus | **-** | **-** | **-** | **-** | **-** | **-** | **-** | **++** | **-** | **-** |
| *Rhombencephalon* |  |  |  |  |  |  |  | | | |
| *Cerebellum* |  |  |  |  |  |  |  | | | |
| Val_gra,_ granular layer of lateral division of valvula cerebelli | **+** | **++** | **-** | **++** | **+** | **++** | **-** | **++** | **-** | **-** |
| Val_mol,_ molecular layer of lateral division of valvula cerebelli | **-** | **-** | **-** | **-** | **-** | **-** | **-** | **-** | **-** | **-** |
| Vam_gra,_ granular layer of medial division of valvula cerebelli | **-** | **++** | **-** | **++** | **+** | **++** | **-** | **++** | **-** | **-** |
| Vam_mol,_ molecular layer of medial division of valvula cerebelli | **-** | **-** | **-** | **-** | **-** | **-** | **-** | **-** | **-** | **-** |
| CCe_gra_, granular layer of corpus cerebellum | **-** | **++** | **-** | **+** | **-** | **+** | **-** | **++** | **-** | **-** |
| CCe_mol_, molecular layer of corpus cerebellum | **-** | **-** | **-** | **-** | **-** | **-** | **-** | **-** | **-** | **-** |
| EG, eminentia granularis | **-** | **++** | **-** | **++** | **-** | **++** | **-** | **++** | **-** | **-** |
| LCa_gra_, granular layer of lobus caudalis cerebelli | **-** | **++** | **-** | **++** | **-** | **++** | **-** | **++** | **-** | **-** |
| LCa_mol_, molecular layer of lobus caudalis cerebelli | **-** | **-** | **-** | **-** | **-** | **-** | **-** | **-** | **-** | **-** |
| *Medulla oblongata* |  |  |  |  |  |  |  | | | |
| brainstem cranial nuclei (except LX) | **-** | **-** | **-** | **-** | **-** | **-** | **-** | **-** | **-** | **-** |
| LX, vagal lobe | **-** | **++** | **-** | **++** | **-** | **++** | **-** | **-** | **-** | **-** |
| Gc, griseum centrale | **-** | **-** | **-** | **-** | **-** | **-** | **-** | **-** | **-** | **-** |
